# A Lox/CHOP‐10 crosstalk governs osteogenic and adipogenic cell fate by MSCs

**DOI:** 10.1111/jcmm.13798

**Published:** 2018-07-25

**Authors:** Wen‐yan Jiang, Chun Xing, Hong‐wei Wang, Wei Wang, Su‐zhen Chen, Liu‐fang Ning, Xu Xu, Qi‐qun Tang, Hai‐yan Huang

**Affiliations:** ^1^ Key Laboratory of Metabolism and Molecular Medicine The Ministry of Education Shanghai China; ^2^ Department of Biochemistry and Molecular Biology School of Basic Medical Sciences Fudan University Shanghai China; ^3^ Biliary and Pancreatic Center Huadong Hospital Fudan University Shanghai China; ^4^ Institute of Stem Cell Research and Regenerative Medicine Institutes of Biomedical Sciences Fudan University Shanghai China

**Keywords:** BAPN, BMP4, CHOP‐10, Lox, obesity, osteoporosis

## Abstract

Accelerated marrow adipogenesis has been associated with ageing and osteoporosis and is thought to be because of an imbalance between adipogenic and osteogenic differentiation of mesenchymal stem cell (MSCs). We have previously found that lysyl oxidase (Lox) inhibition disrupts BMP4‐induced adipocytic lineage commitment and differentiation of MSCs. In this study, we found that lox inhibition dramatically up‐regulates BMP4‐induced expression of CCAAT/enhancer binding protein (C/EBP) homologous protein 10 (CHOP‐10), which then promotes BMP4‐induced osteogenesis of MSCs both in vitro and in vivo. Specifically, Lox inhibition or CHOP‐10 up‐regulation activated Wnt/β‐catenin signalling to enhance BMP4‐induced osteogenesis, with pro‐adipogenic p38 MAPK and Smad signalling suppressed. Together, we demonstrate that Lox/CHOP‐10 crosstalk regulates BMP4‐induced osteogenic and adipogenic fate determination of MSCs, presenting a promising therapeutic target for osteoporosis and other bone diseases.

## 1 INTRODUCTION

Bone is a pivotal tissue that provides structural support and physical protection to various organs within the body.^1^, ^2^ Bone dysfunction is associated with diverse conditions, such as osteoporosis, fracture and rheumatoid arthritis.^1^ Bone is also highly dynamic and is constantly remodelled by an orchestrated balance between osteoclastic bone resorption and osteoblastic bone formation.^3^ However, an increase in marrow adipose tissue content and decreased bone volume have also been observed in osteoporosis, diabetes and ovariectomy because of an imbalance between adipogenesis and osteogenesis from mesenchymal stem cells (MSCs).^4^ Furthermore, there is evidence that this unbalanced proadipocytic and anti‐osteoblastic MSCs allocation could result from inhibition of the TGF‐β/BMP and Wnt/β‐catenin signalling pathways.^3^, ^5^

Bone morphogenetic proteins (BMPs) are extracellular cytokines originally isolated from bone extracts and have been found to induce ectopic chondrogenesis and osteoblastogenesis.^6^ It has been reported that the BMP‐2, BMP‐4, BMP‐6, BMP‐7, and BMP‐9 subtypes promote osteogenic commitment, as well as terminal osteogenic differentiation in MSCs.^7^ Additionally, recombinant human BMP‐2 and BMP‐7 have been clinically applied in various bone disorders.^6^ However, it has also been demonstrated that BMPs have pro‐adipogenic effects.^8^ For example, BMP2/4 can induce adipocytic lineage commitment of MSCs through activation of p38 Smad/MAPK signalling.^9^, ^10^, ^11^ In addition, other side effects, such as increased cancer risk related to higher BMP doses, have also been reported.^12^ The above studies indicate that enhancing pro‐osteogenic and blocking pro‐adipogenic effects with a lower dose of BMPs may be effective for clinical use of BMPs in bone disorders.

Lysyl oxidase (Lox) is synthesized and secreted as a 50 kD pro‐enzyme (Pro‐Lox) into the extracellular environment followed by enzymatic cleavage yielding the 32 kD mature and active Lox enzyme (Ma‐Lox) and the 18 kD pro‐peptide (Lox‐PP).^13^ The mature, 32 kD Lox catalyses the cross‐linking of elastin and collagen, which is indispensable for the structural integrity and function of bone tissue.^14^ We have previously reported that Lox promotes BMP2/4‐induced adipocytic lineage commitment of C3H10T1/2 MSCs and that knockdown of Lox disrupts this commitment process.^10^ Therefore, based on the theoretical inverse balance between adipogenic and osteogenic programming,^6^ it raises a question whether Lox inhibition would promote BMP4‐induced osteogenesis of MSCs. Here, we discovered that Lox inhibition promotes the osteogenic fate decisions of MSCs by modulating the expression of CCAAT/enhancer binding protein (C/EBP) and homologous protein 10 (CHOP‐10), with pro‐osteogenic Wnt/β‐catenin signalling activation. Therefore, our studies open up opportunities for novel therapeutic intervention in bone diseases.

## 2 MATERIALS AND METHODS

### 2.1 Animal studies

Mice were housed in a controlled environment (12 hours light/dark cycle, 60%‐70% humidity). For high‐fat diet‐induced obesity and osteoporosis, 6‐week‐old male C57BL/6J mice were fed a high‐fat diet (HFD, SLACOM, 51% kcal from fat) for 10 months, with mice fed a normal chow diet (NCD, SLACOM, 10% kcal from fat) used as controls. To examine the effect on bone of Lox inhibition when combined with elevated levels of BMP4 in vivo*,* we used previously described Fabp4‐driven, male BMP4‐Tg mice,^15^ which also constitutively overexpress BMP4 in bone marrow adipose tissue, providing a higher concentration of BMP4 in local bone environment. We screened the mice by PCR using primers (Fabp4‐BMP4 tg: cagtgatcattgccagggagaacc; gcctcctagcaggacttggcta), control mice were non‐Tg littermates. In this study, to avoid the influence of oestrogen on bone formation,^16^, ^17^ only the male mice was used. For β‐aminopropionitrile (BAPN, Sigma‐Aldrich, St. Louis, MO, USA) administration, 6‐week‐old male BMP4‐Tg mice were daily injected ip with BAPN (100 mg/kg/d) or PBS control for 2 weeks.^18^ Following treatment, the right femurs of mice were subjected to micro‐CT analysis. For all in vivo experiments, 3‐5 technical replicates were performed in each independent experiment. All animal experiments were approved by the Animal Care and Use Committee of Fudan University Shanghai Medical College and followed the National Institute of Health guidelines on the care and use of animals.

### 2.2 Cell culture and induction of commitment/differentiation

Inguinal white adipose tissue (iWAT) was obtained from 6‐ to 8‐week‐old male C57BL/6J mice. Fat pads were minced and digested for 40 minutes at 37°C (1 mg/mL Collagenase IV (Sigma‐Aldrich, St. Louis, MO, USA) in DMEM). The cell suspension was passed through a 100‐μm filter and centrifuged at 500 × *g* for 5 minutes at 4°C. The SVF pellets were resuspended in F12/DMEM with 10% foetal bovine serum (FBS). C3H10T1/2 mesenchymal stem cells were cultured in DMEM containing 10% calf serum. Primary bone marrow stromal cells (BMSCs) isolated from 6‐week‐old male C57BL/6J mice were cultured in a fresh alpha‐minimum essential medium (α‐MEM) containing 10% FBS. When BMSCs reach 80%‐90% confluent, they were passaged and used in the experiments below.

To induce lineage commitment, SVFs or C3H10T1/2 cells were seeded at 30% confluence and cultured with or without purified recombinant BMP4 (10 ng/mL) until 2‐day post‐confluence (day 0). To induce adipocyte differentiation, C3H10T1/2 cells or SVF cells (day 0) were treated according to a previously described protocol (MDI), with Oil Red O staining conducted to detect lipid droplets.^10^ To induce osteoblast differentiation, SVF or C3H10T1/2 cells (day 0) were cultured in F12/DMEM or DMEM with 10% FBS, 10 nmol/L dexamethasone, 0.2 mmol/L L‐ascorbic acid and 10 mmol/L β‐glycerophosphate. To induce osteoblast differentiation of BMSCs, BMSCs were cultured in α‐MEM with 10% FBS, 0.2 mmol/L L‐ascorbic acid and 10 mmol/L β‐glycerophosphate. Alizarin Red S staining was then used to detect any calcium deposits.

### 2.3 BAPN/IWR‐1‐endo treatment

C3H10T1/2 stem cells were seeded at 30% confluence and cultured in DMEM containing 10% calf serum both with and without purified recombinant BMP4 until 2‐day post‐confluence (day 0). BAPN (200 μmol/L, Sigma‐Aldrich, St. Louis, MO, USA) or IWR‐1‐endo (5 μmol/L, SelleckChem, Houston, TX, USA) was daily added from the beginning culture to 2‐day post‐confluence.

### 2.4 RNA interference

Stealth siRNA duplexes specific for mouse Lox were designed and synthesized by Invitrogen. The sequence for successful Lox RNAi knockdown was GCGGAUGUCAGAGACUAUGACCACA.^10^ Stealth siRNA‐negative control duplexes with similar GC content were used as control. SVF or C3H10T1/2 cells were transfected at 30%~50% confluence with siRNA oligonucleotides using Lipofectamine RNAi MAX according to the manufacturer's instructions (Invitrogen, Carlsbad, CA, USA).

### 2.5 Western blotting

Both cell and tissue extracts were generated using lysis buffer containing 50 mmol/L Tris–HCl (pH 6.8), 2% SDS, 100 mmol/L NaF, 1 mmol/L PMSF and a phosphatase and protease inhibitor mixture (Roche Applied Science, Indianapolis, IN, USA). Equal amounts of protein were subjected to SDS‐PAGE and immunoblotted with specific primary antibodies. Primary antibodies were as follows: Lox, CHOP‐10, Hsp90, (Santa Cruz, Delaware Ave, CA, USA); Runx2 (MBL, Nagoya, Japan); Osterix (Abcam, Cambridge, UK); Osteocalcin (Ocn) (Millipore, Billerica, MA, USA); Col1α1 (Sigma‐Aldrich); Axin, GSK3β, phospho‐GSK3β, PPARγ (Cell Signaling Technology, Beverly, MA, USA); β‐catenin (Enogene, New York, NY, USA); 422/aP2 was provided by Dr. M Daniel Lane.

### 2.6 Plasmid construction

MSCV‐mature Lox was generated as previously described.^19^ The MSCV‐CHOP‐10 expression plasmid was generated using standard DNA cloning techniques. Briefly, the mouse cDNA for CHOP‐10 was amplified and subsequently cloned into the pMSCV‐puro retroviral vector between the XhoI (5′‐end) and EcoRI (3′‐end) restriction sites using the following primers: 5′‐CctcgagGATGGCAGCTGAGTCCCTGCCTTTCACCT‐3′(forward), 5′‐GgaattcCTCATGCTTGGTGCAGGCTGACCAT‐3′(reverse).

### 2.7 Q‐PCR

Total RNA was isolated using Trizol reagent (Thermo Fisher Scientific, Waltham, MA, USA) and reverse transcribed into cDNA using PrimeScript RT Master Mix (TaKaRa, Dalian, China). The mRNA levels of the investigated genes were measured using SYBR Green Master Mix by 7500 Real‐time PCR (Applied Biosystems, Foster City, CA, USA). The genes investigated and the primers used are listed in Table 1.

**Table 1:** Primers for Q‐PCR

Primers	Sequence	
18S rRNA	Forward	5′‐CGGCTACCACATCCAAGGAA‐3′
	Reverse	5′‐GCTGGAATTACCGCGGCT‐3′
CHOP‐10	Forward	5′‐CTGCCTTTCACCTTGGAGAC‐3′
	Reverse	5′‐CGTTTCCTGGGGATGAGATA‐3′
Lox	Forward	5′‐ ACTTCCAGTACGGTCTCCCG‐3′
	Reverse	5′‐ GCAGCGCATCTCAGGTTGT‐3′
Runx2	Forward	5′‐ TTACCTACACCCCGCCAGTC‐3′
	Reverse	5′‐TGCTGGTCTGGAAGGGTCC‐3′
Osterix	Forward	5′‐ ATGGCGTCCTCTCTGCTTG‐3′
	Reverse	5′‐TGAAAGGTCAGCGTATGGCTT‐3′
Ocn	Forward	5′‐ GGGCAATAAGGTAGTGAACAG
	Reverse	3′‐ GCAGCACAGGTCCTAAATAGT
Col1α1	Forward	5′‐ GCTCCTCTTAGGGGCCACT‐3′
	Reverse	5′‐CCACGTCTCACCATTGGGG‐3
Wnt1	Forward	5′‐ TTACCTACACCCCGCCAGTC‐3′
	Reverse	5′‐TGCTGGTCTGGAAGGGTCC‐3′
Wnt3a	Forward	5′‐ TGCTGTTGAGGCAATGGTC‐3′
	Reverse	5′‐CAGATGGGCTGTATGTA‐3′
Wnt5a	Forward	5′‐ CAACTGGCAGGACTTTCTCAA ‐3′
	Reverse	5′‐ CATCTCCGATGCCGGAACT ‐3
Wnt10a	Forward	5′‐ GCTCAACGCCAACACAGTG ‐3′
	Reverse	5′‐ CGAAAACCTCGGCTGAAGATG ‐3′
Dkk1	Forward	5′‐ CTCATCAATTCCAACGCGATCA ‐3′
	Reverse	5′‐ GCCCTCATAGAGAACTCCCG ‐3′

### 2.8 Oil Red O staining

C3H10T1/2 stem cells were induced to adipocyte differentiation as described above. On day 8, the cells were washed three times with PBS (phosphate‐buffered saline) and then fixed for 10 minutes with 3.7% formaldehyde. Oil Red O (0.5% in isopropyl alcohol) was diluted with water (3:2), filtered through a 0.45‐μm filter and incubated with the fixed cells for 1 hour at room temperature. The cells were then washed with water, and the stained fat droplets in the adipocytes were visualized by light microscopy and photographed.

### 2.9 Alizarin Red S staining

C3H10T1/2 stem cells, BMSCs or SVFs from inguinal adipose tissue were induced to osteoblast differentiation as described above. On day 8 or 14, the cells were washed three times with PBS (phosphate‐buffered saline) and then fixed for 10 minutes with 3.7% formaldehyde. Alizarin Red S solution (1% in 0.1 mol/L Tris–HCl, pH9.0) was filtered through a 0.45 μm filter and incubated with the fixed cells for 1 hour at room temperature. The cells were then washed with water, and the stained mineralization in the osteoblasts was visualized by light microscopy and photographed.

### 2.10 In vivo bone formation assay

C3H10T1/2 cells were treated as described above and then cultured in the above‐mentioned osteogenic differentiation medium for 3 days. Approximately 4 × 10^6^ cells were mixed with 40 mg hydroxyaptite‐tricalcium phosphate (HA‐TCP) powders (Sigma‐Aldrich) and subcutaneously implanted into the armpit of 6‐week‐old BALB/c nude mice (n = 4). Implants were harvested after 4 weeks.

### 2.11 Micro‐CT

The 4% paraformaldehyde‐fixed femurs were subjected to micro‐CT analysis. Bone parameters of the femur metaphysis were quantified ex vivo using high‐resolution, X‐ray micro‐computed tomography (Quantum GX micro‐CT, PerkinElmer, Boston, MA, USA). The bone parameters included bone volume fraction (BV/TV), trabecular thickness (Tb.Th), trabecular number (Tb.N) and trabecular separation (Tb.Sp). Additionally, cortical area (Ct.Ar), total tissue area (Tt.Ar), cortical area/total tissue area (Ct.Ar/Tt.Ar) and cortical thickness (Ct.Th) were also measured. Beginning 2 mm distal to the growth plate, metaphysis was evaluated for trabecular and cortical bone at 4.5‐μm resolution and 90 keV intensity settings. A threshold for each slice was set exclusively for cortical and trabecular bone according to the manufacturer's instructions. The reconstructed 3D images were then used to quantify bone micro‐architecture.

### 2.12 Statistical analysis

Results were expressed as mean ± SEM. Comparisons between groups were determined by Student's *t* test, or ANOVA. *P *<* *0.05 was considered statistically significant. All experiments were repeated at least three times, and representative data are shown.

## 3 RESULTS

### 3.1 Lox inhibition enhances BMP4‐induced osteogenesis

**Figure 1:**
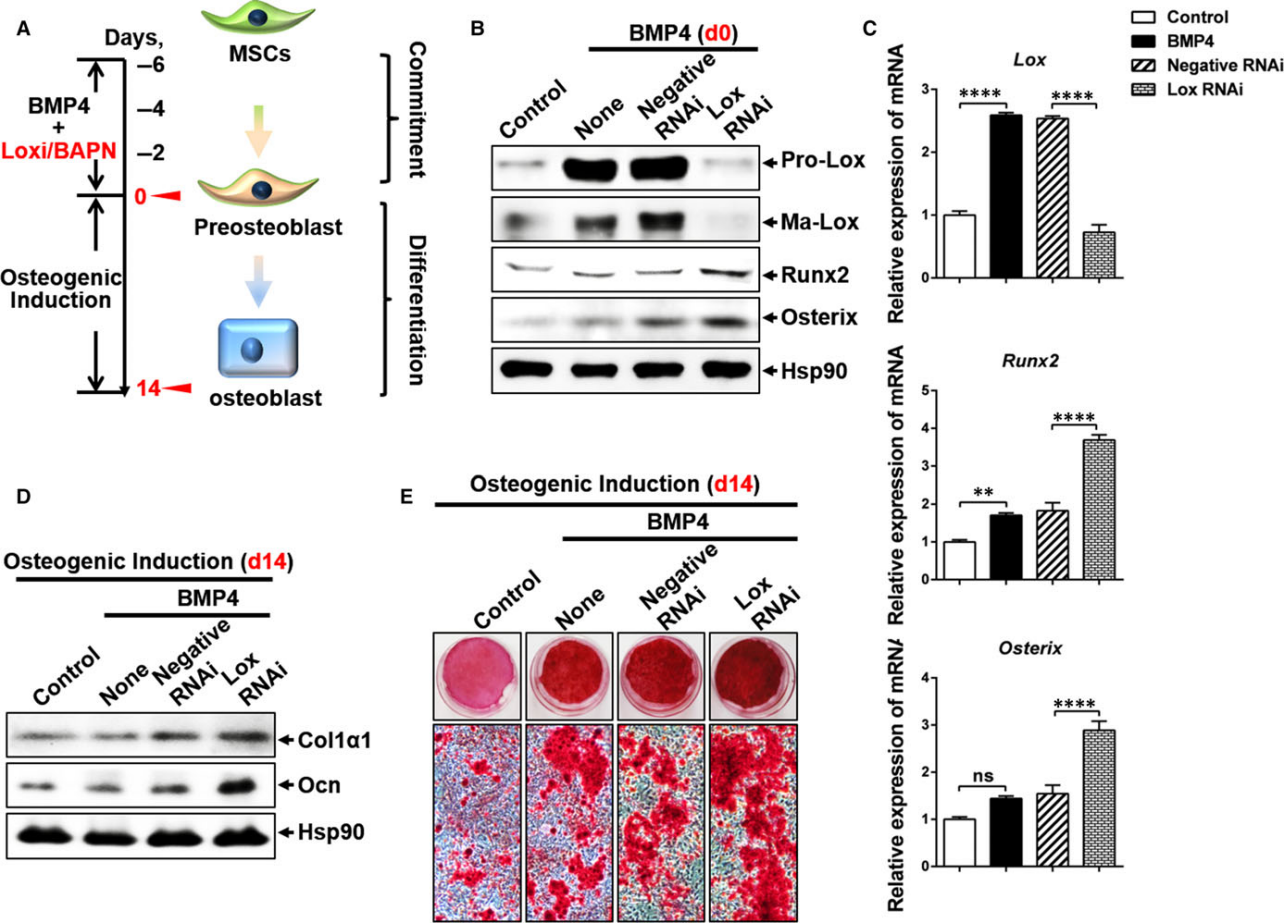
Lox inhibition enhances BMP4‐induced osteogenesis. A, Diagram for osteoblastic lineage commitment and subsequential differentiation by MSCs. C3H10T1/2 cells were seeded at 30% confluence and cultured in DMEM containing 10% calf serum, with or without purified recombinant BMP4 (10 ng/mL) until 2‐day post‐confluence (day 0). To induce osteoblast differentiation, cells (day 0) were cultured in DMEM with 10% FBS, 10 nmol/L dexamethasone, 0.2 mmol/L L‐ascorbic acid and 10 mmol/L β‐glycerophosphate for 14 d. B, C, The effects of Lox knockdown on BMP4‐induced osteoblast lineage commitment (day 0) indicated by Runx2 and Osterix were assessed by Western blotting (B) and Q‐PCR (C). D, E, The effects of Lox knockdown on BMP4‐induced osteogenic differentiation (day 14) was evaluated by Western blotting (D) and Alizarin Red S staining (E)

Several studies have demonstrated that BMP4 has a well‐established role in triggering commitment of mesenchymal stem cells into the osteogenic and adipogenic lineage.^8^, ^9^, ^10^, ^15^ In addition, BMP4‐regulated osteogenic and adipogenic lineage commitment of MSCs is mutually exclusive.^8^ Based on our previous studies that knockdown of Lox disrupts the BMP4‐induced adipocyte lineage commitment from MSCs^10^ and a theoretical inverse relationship exists between osteogenic and adipogenic lineage commitment and differentiation of MSCs,^6^ we hypothesized that knockdown of Lox would enhance BMP4‐induced osteogenesis of MSCs. To explore this, C3H10T1/2 cells, a well‐characterized mesenchymal stem cells^20^ and faithful model of MSCs to study adipocytic and osteogenic commitment and differentiation both in vitro and in vivo,^8^, ^9^, ^21^ was used. As expected, two key osteogenic transcription factors, Runx2 and Osterix, were up‐regulated by BMP4, which were further enhanced by Lox knockdown at the committed stage (day 0) (Figure 1A‐C). The 2‐day post‐confluent C3H10T1/2 cells were then subjected to the osteogenic culture medium to induce osteogenesis. Correspondingly, BMP4 treatment increased the expression of osteogenic markers Col1α1 and Ocn and calcium deposition after osteogenic induction (Figure 1D,E), which was further elevated by Lox knockdown. We next investigated whether inhibition of Lox enzymic activity with β‐aminopropionitrile (BAPN)^18^ would influence osteogenic differentiation of C3H10T1/2 cells. Similarly, BAPN enhanced BMP4‐induced osteogenic lineage commitment with increased expression of Runx2 and Osterix at committed stage (Figure S1A), and induced higher expression of Col1α1 and Ocn and more calcium deposition after osteogenic induction (Figure S1B,C). Besides, similar results were also observed in stromal vascular fractions (SVFs) from mice inguinal adipose tissue (Figure S1D‐J) and BMSCs (Figure S1K,L). However, we also found BAPN alone inhibited osteogenesis both in vitro (Figure S1) and in vivo (Figure S5) which was consistent with the previous studies that Lox inhibit osteoblast differentiation.^14^, ^22^ This discrepancy of Lox function might be caused by the presence of BMP4. These results collectively demonstrated that inhibition of Lox expression or its enzymic activity enhances BMP4‐induced osteogenesis by MSCs.

### 3.2 CHOP‐10 up‐regulation contributes to the enhanced osteogenic lineage commitment by Lox inhibition

**Figure 2:**
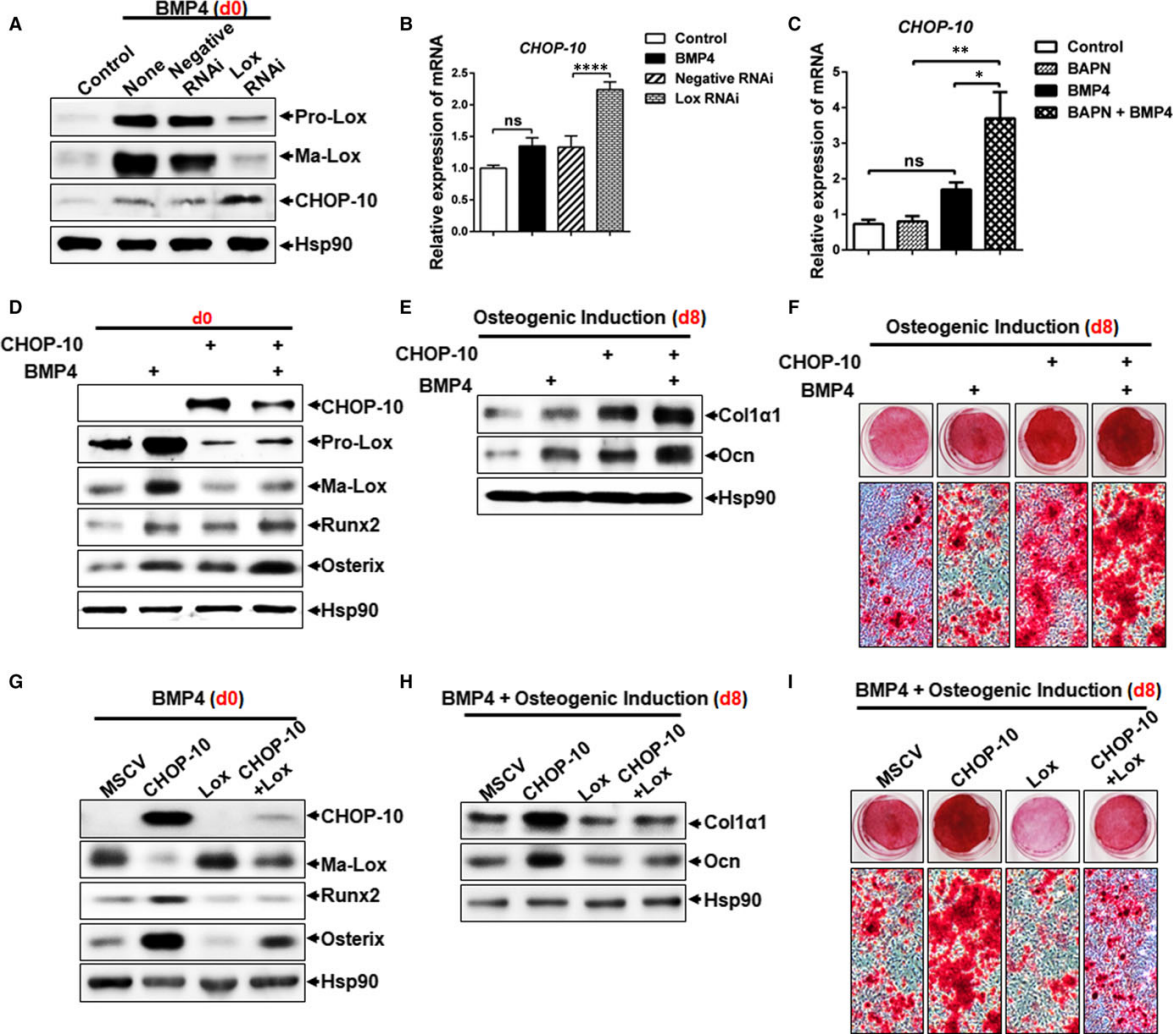
CHOP‐10 up‐regulation contributes to the enhanced osteogenic lineage commitment by Lox inhibition. A, B, The effect of Lox knockdown on CHOP‐10 expression in C3H10T1/2 at the committed stage (day 0) was assessed by Western blotting (A) and Q‐PCR (B). C, The effect of the Lox inhibitor BAPN on the expression of CHOP‐10 in C3H10T1/2 at the committed stage was confirmed by Q‐PCR. D, Osteoblast‐specific markers at the committed stage were detected by Western blotting. E, Expression of Col1α1 and Ocn on day 8 after osteogenic induction. F, Calcium deposition stained by Alizarin Red S on day 8 after osteogenic induction. G, Expression of Runx2 and Osterix at the committed stage (day 0). H, I, Expression of Col1α1, Ocn (H), and calcium deposits stained by Alizarin Red S (I) on day 8 after osteogenic induction

It has been previously reported that CHOP‐10 inhibits terminal adipocyte differentiation in 3T3‐L1 preadipocytes^23^ and enhances osteoblastic differentiation in ST‐2 stromal cells.^24^ We next explored whether CHOP‐10 participated in the enhancement of BMP4‐induced osteogenesis mediated by Lox inhibition. Our results showed that BMP4 induced CHOP‐10 expression in C3H10T1/2 cells, and knockdown of Lox expression or inhibition of Lox enzymic activity with BAPN further up‐regulated CHOP‐10 expression both in mRNA and in protein levels (Figures 2A‐C and S2A). Additionally, similar results were also observed in BMSCs (Figure S1K‐L) and SVF cells from mice inguinal adipose tissue (Figure S2B‐E). To test whether up‐regulation of CHOP‐10 inhibited BMP4‐induced osteogenic lineage commitment, CHOP‐10 was overexpressed in C3H10T1/2 cells using a retroviral construct. Compared to the control cells, Runx2 and Osterix were dramatically increased in cells overexpressing CHOP‐10 and further enhanced by BMP4 treatment at the committed stage (Figures 2D and S3A). Furthermore, when induced in osteogenic medium, CHOP‐10 overexpressing cells exhibited significantly increased Col1α1 and Ocn expression (Figure 2E), along with greater calcium deposition, which was further enhanced by BMP4 (Figure 2F). The pro‐osteogenic effect of CHOP‐10 was at the expense of adipocyte phenotype, as indicated by decreased expression of PPARγ and 422/aP2, as well as decreased Oil Red O staining (Figure S3B‐D). Notably, CHOP‐10 overexpression also suppressed the expression of both pro‐ and mature Lox at the committed stage (Figure S3B). These findings demonstrate that Lox/CHOP‐10 crosstalk regulates the balance between osteogenesis and adipogenesis. In line with these results, forced expression of mature Lox partially inhibited enhanced osteogenesis by CHOP‐10 overexpression, as indicated by decreased Runx2 and Osterix expression at the committed stage (Figure 2G), and reduced Col1α1 and Ocn expression (Figure 2H),and lower calcium deposition when transferred to an osteogenic culture medium (Figure 2I). Consistently, increased expression of specific adipocyte marker and greater lipid droplets were observed in cells co‐overexpressing CHOP‐10 and mature Lox than those in cells overexpressing CHOP‐10 alone (Figure S3E,F).

### 3.3 Wnt/β‐catenin signalling is involved in the enhanced osteogenesis by Lox inhibition or CHOP‐10 overexpression

**Figure 3:**
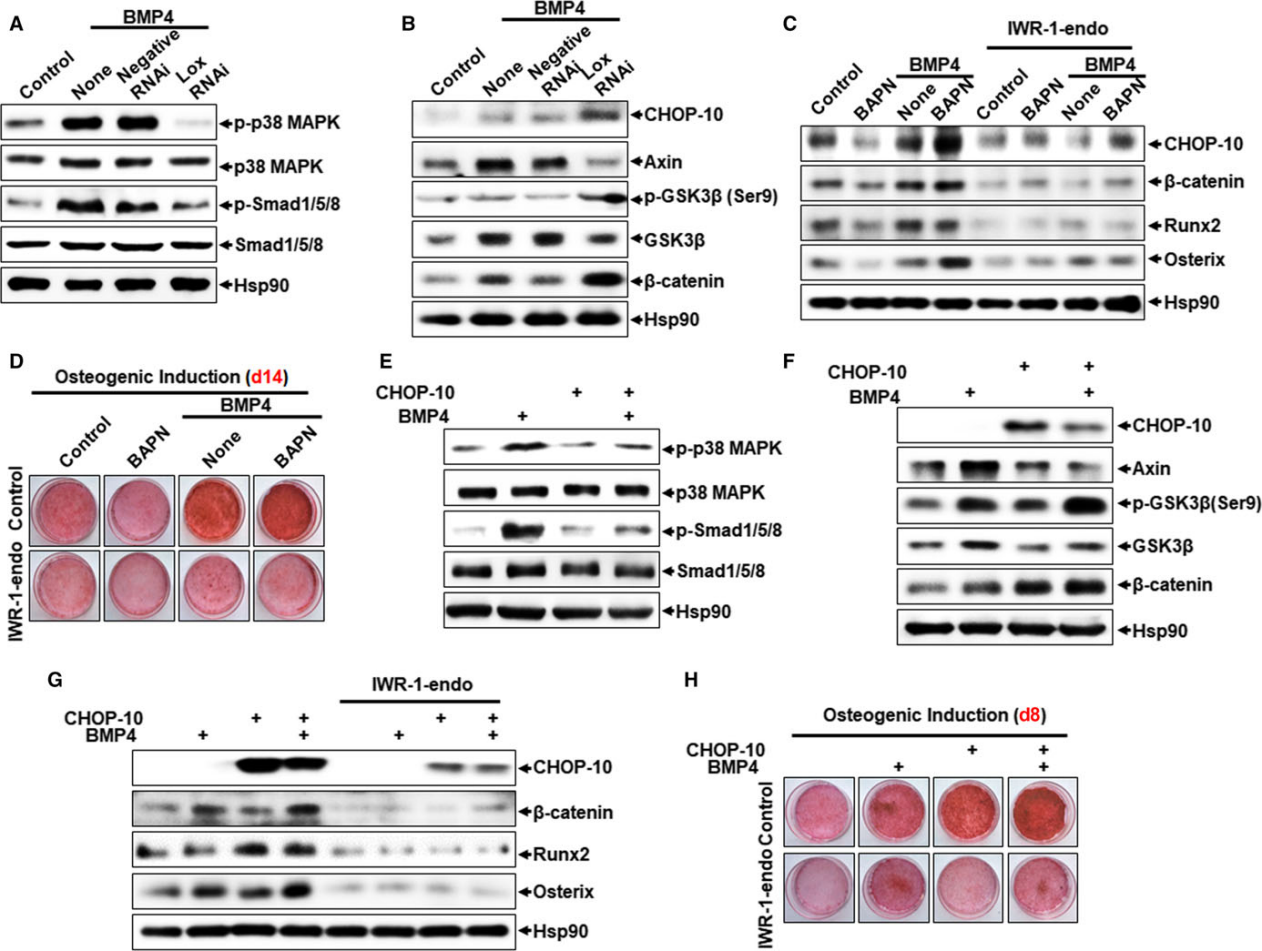
Signalling pathway involved in enhanced BMP4‐induced osteogenesis by Lox inhibition or CHOP‐10 overexpression. A, The effect of Lox knockdown on p38 MAPK and Smad signalling. B, The effect of Lox knockdown on Wnt/β‐catenin signalling. C, D, The effect of IWR‐1‐endo (5 μmol/L) on Lox inhibition enhanced osteogenic lineage commitment and subsequential differentiation. E, The effect of CHOP‐10 overexpression on p38 MAPK and Smad signalling. F, The effect of CHOP‐10 overexpression on Wnt/β‐catenin signalling. G, H, The effect of IWR‐1‐endo (5 μmol/L) on CHOP‐10 overexpression enhanced osteogenic lineage commitment and subsequential differentiation

Next, we investigated the molecular mechanisms by which Lox and CHOP‐10 regulates unbalanced adipogenesis and osteogenesis. We previously reported that BMP4 activates Smad and p38 MAPK signalling to induce Lox, which promotes adipocyte lineage commitment.^10^ Here we found that Lox inhibition decreased phosphorylation of Smad1/5/8 and p38 MAPK induced by BMP4 (Figure 3A). In contrast, the Wnt/β‐catenin signalling pathway, which is central to bone development and homoeostasis,^3^, ^25^ was greatly activated by Lox inhibition (Figure 3B). We showed that Lox inhibition increased GSK‐3β phosphorylation at Ser 9 and up‐regulated β‐catenin, while Axin and total GSK‐3β were both decreased (Figure 3B), suggesting higher Wnt activity by Lox inhibition through negative regulation of destructive complexes. Wnt ligands promoting osteogenesis, such as *Wnt1*,* Wnt3a*,* Wnt5a* and *Wnt10a*, were also elevated by Lox inhibition in SVFs from mice inguinal adipose tissue (Figure S4). While the Wnt inhibitor *Dkk1* was down‐regulated, Wnt effector *Tcf4* was increased by Lox inhibition (Figure S4). It has been previously demonstrated that IWR‐1‐endo, an inhibitor of Wnt pathway, induces Axin2 protein levels and promotes β‐catenin degradation by stabilizing Axin‐scaffolded destructive complexes.^26^ Here, we found that IWR‐1‐endo treatment decreased protein levels of β‐catenin and CHOP‐10 expression induced by Lox inhibition, while subsequently repressing osteogenesis with lower expression of Runx2 and Osterix, along with lower calcium deposition (Figure 3C,D).

Similarly, CHOP‐10 overexpression enhanced both basal and BMP4‐induced Wnt/β‐catenin signalling, with pro‐adipogenic p38 MAPK and Smad signalling suppressed (Figure 3E,F), while IWR‐1‐endo treatment blocked CHOP‐10‐induced osteogenesis as indicated by decreased expression of Runx2 and Osterix and less Alizarin Red S staining (Figure 3G,H). These results show that both Lox inhibition and CHOP‐10 overexpression promote BMP4‐induced osteogenesis via activation of pro‐osteogenic Wnt/β‐catenin signalling and inactivation of pro‐adipogenic Smad and p38 MAPK signalling.

### 3.4 Lox inhibition enhances bone formation in vivo

**Figure 4:**
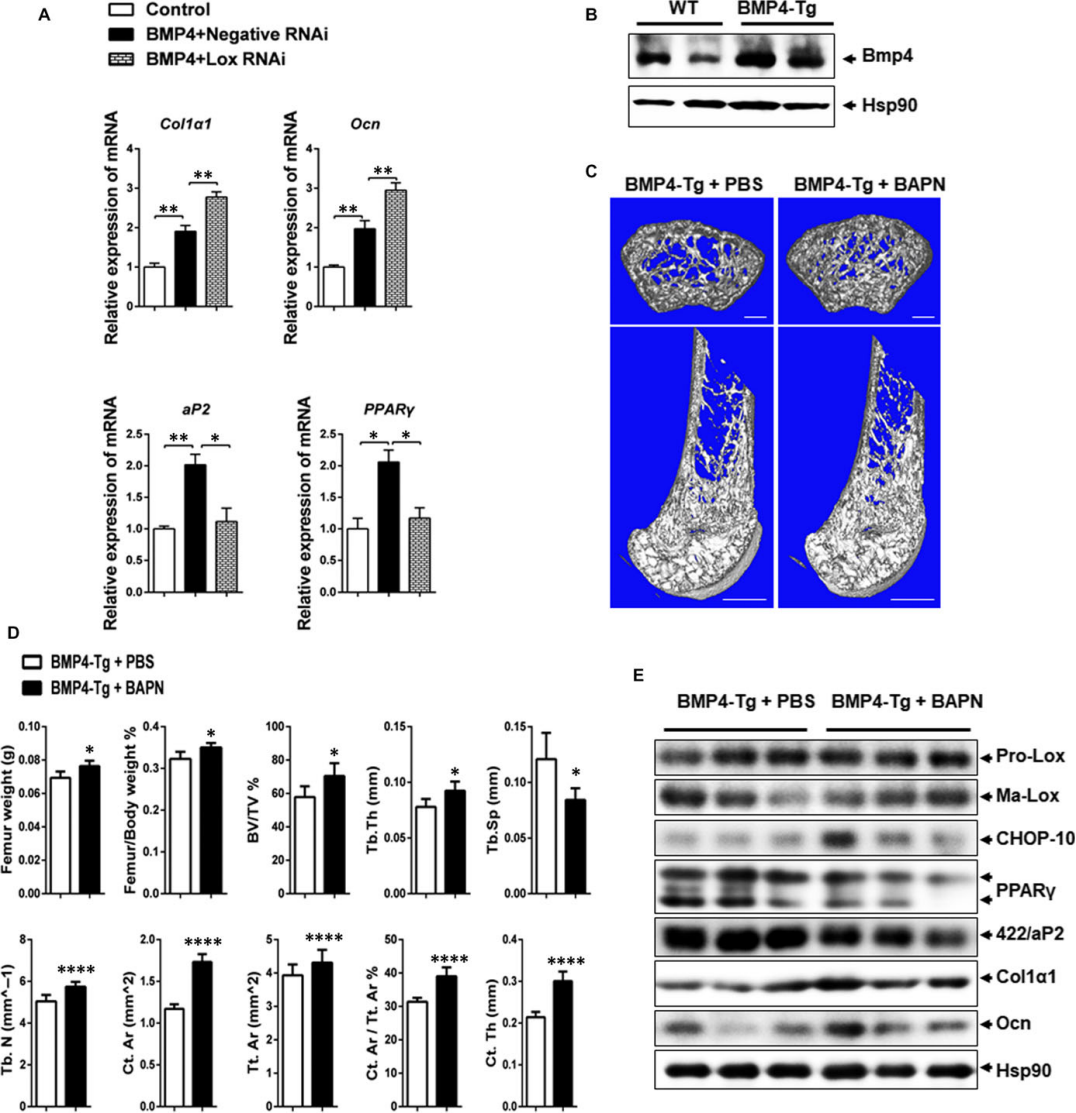
Lox inhibition together with BMP4 enhances bone formation in vivo. A, C3H10T1/2 stem cells transfected with Lox RNAi or negative RNAi in the presence of BMP4 were transplanted with HA‐TCP (hydroxyaptite‐tricalcium phosphate) subcutaneously into the armpit of immunocompromised mice for 4 wk. The transplants were harvested. Expression of osteogenic and adipogenic markers in transplants was evaluated by Q‐PCR (n = 4). B, BMP4 expression in the femur from WT and BMP4‐Tg mice. C, 3D micro‐CT images of cross sections (upper panel) and vertical sections (lower panel) of right femur extracted from BMP4‐Tg mice with PBS or BAPN treatment. Bars: upper panel, 200 μm; lower panel, 1 mm. D, Quantification of trabecular and cortical bone for the same femur as C. Results are shown as mean ± SEM. E, Western blotting of Lox, CHOP‐10, PPARγ, 422/aP2, Col1α1 and Ocn expression in the right femur extracted from BMP4‐Tg mice with PBS or BAPN treatment. *P <* *0.05, **P <* *0.01, ***P <* *0.001, ****P <* *0.0001

The above results have shown that Lox inhibition enhances BMP4‐induced osteogenesis in vitro. As we mentioned earlier, C3H10T1/2 cell line behaves similarly to mesenchymal stem cells,^20^ making this cell line ideal for studying adipocytic and osteogenic commitment and differentiaotion both in vitro and in vivo. We next investigated the bone formation capacity of C3H10T1/2 cells with Lox inhibition in the presence of BMP4 in vivo. To which, these cells were induced to undergo commitment in cell culture and then implanted s.c. into athymic mice, conditions under which they give rise to tissue that is indistinguishable from endogenous tissue.^8^, ^9^, ^21^ As expected, the expression of osteogenic markers Col1α1 and Ocn was increased in the Lox RNAi group, while adipogenic markers PPARγ and 422/aP2 were decreased (Figure 4A).

Next, we investigated whether Lox inhibition in conjunction with BMP4 could enhance bone formation in vivo. Here, we use Fabp4‐driven male BMP4‐Tg to do the experiment for the following reason. On the one hand, BMP4 is a secretory protein and has an endocrine effect.^27^ Therefore, BMP‐4 overexpressed by the adipose tissues might be released into serum and reach all tissues including bone. On the other hand, BMP4 might be also overexpressed in bone of Fabp4‐driven BMP4‐Tg mice owing to the presence of Fabp4 in bone marrow adipose tissue (BMAT). To confirm this, we have tested the expression of BMP4 in bone of Fabp4‐driven BMP4‐Tg mice, which was showed that BMP4 was indeed elevated in the bone of Fabp4‐driven BMP4‐Tg mice (Figure 4B). Therefore, the local BMP4 in bone is constitutively overexpressed in Fabp4‐driven BMP4‐Tg owing to both the paracrine and endocrine function of BMP4 from marrow adipose tissue and other adipose tissue.

The 6‐week‐old BMP4‐Tg mice were injected ip with BAPN (1 mg/kg/d) or PBS control daily for 2 weeks. The femurs were then stripped and analysed by micro‐CT. Analysis of both cross and vertical femur sections illustrated that BAPN treatment enhanced bone formation, resulting in more trabeculae than that found in PBS controls (Figure 4C). The BAPN‐treated femurs displayed increased bone volume fraction (BV/TV), trabecular thickness (Tb.Th) and trabecular number (Tb.N), along with decreased trabecular separation (Tb.Sp). In addition, cortical area (Ct.Ar), total tissue area (Tt.Ar), cortical area/total tissue area (Ct.Ar/Tt.Ar) and cortical thickness (Ct.Th) were also increased in the BAPN treated group as compared to PBS controls (Figure 4D). Consistently, the expression of adipocyte markers PPARγ and 422/aP2 was decreased, while the osteogenic markers Col1α1 and Ocn were increased in the BAPN treated femurs (Figure 4E). However, decreased bone formation was observed in wild‐type mice with BAPN injection (Figure S5). Therefore, we have concluded that Lox inhibition, together with BMP4, enhances bone formation in vivo.

### 3.5 Lox is up‐regulated in obesity‐induced bone loss

**Figure 5:**
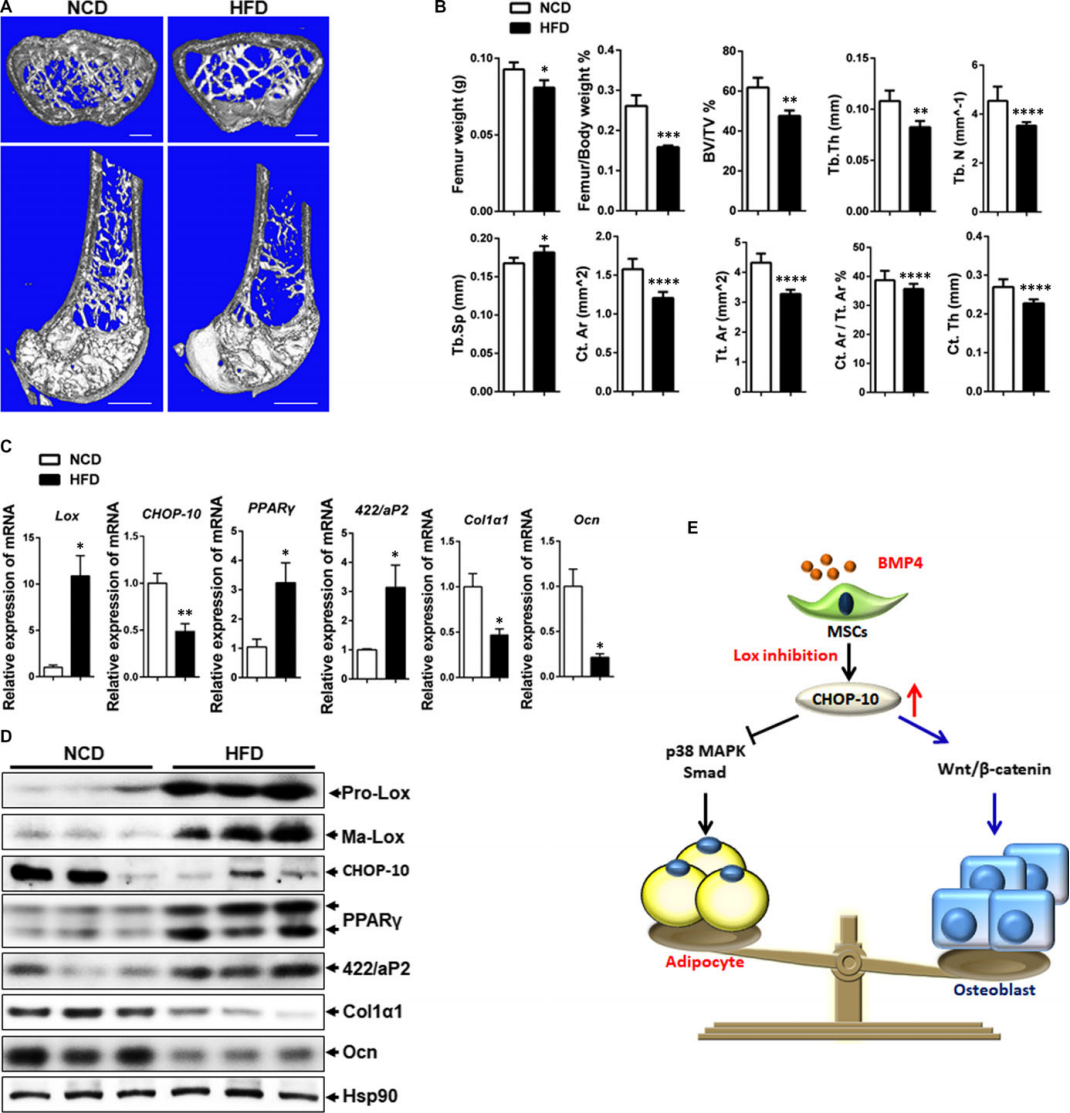
High‐fat diet mice displayed bone loss accompanied with Lox induction. A, 3D micro‐CT images of cross sections (upper panel) and vertical sections (lower panel) of right femur extracted from NCD mice or HFD mice. Bars: upper panel, 200 μm; lower panel, 1 mm. B, Quantification of trabecular and cortical bone for the same femur as shown in Figure 1A. C, Q‐PCR analysis of Lox, PPARγ, 422/aP2, Col1α1 and Ocn expression in femurs of NCD (n = 5) and HFD (n = 5) mice. D, Western blotting of Lox, PPARγ, 422/aP2, Col1α1 and Ocn expression in femurs of NCD (n = 3) and HFD (n = 3) mice. NCD, normal chow diet; HFD, high‐fat diet. E, Graphical abstract. *P <* *0.05, **P <* *0.01, *** *P *<* *0.001, ****P <* *0.0001

It has been demonstrated that male C57BL6 mice fed a high‐fat diet was associated with a reduced rate of bone formation and turnover^28^ by increasing the number of lineage committed adipogenic progenitors in murine marrow at the expense of osteogenic lineage commitment,^29^ which potentially explained the association between increased marrow adipose tissue and increased fracture risk in diseases such as osteoporosis.^30^, ^31^, ^32^ To determine the relationship between Lox and CHOP‐10 in vivo, HFD‐induced obesity and osteoporosis model was used in mice. Micro‐CT analysis revealed fewer trabeculae in the femur metaphysis of HFD than NCD mice (Figure 5A,B). HFD mice also showed remarkably decreased BV/TV, Tb.Th and Tb.N, along with increased Tb.Sp. Additionally, Ct.Ar, Tt.Ar, Ct.Ar/Tt.Ar and Ct.Th were also decreased in HFD mice (Figure 5B). Enhanced adipogenesis and repressed osteogenesis were also consistently observed in the femur of HFD mice as indicated by increased expression of the adipocyte markers PPARγ and 422/aP2, and lower expression of the osteogenic markers Col1α1 and Ocn (Figure 5C,D), with an opposite pattern displayed in NCD mice. These results demonstrate a significant bone loss in HFD‐induced obesity and osteoporosis mice. Accordingly, both pro‐adipogenic pro‐Lox and mature Lox were obviously induced in the femur of HFD mice, with pro‐osteogenic CHOP‐10 expression repressed (Figure 5C,D). Collectively, these results demonstrate a significant bone loss in HFD mice, with increased Lox expression and decreased CHOP‐10 expression in bone, suggesting the potential role of Lox and CHOP‐10 in bone homoeostasis.

## 4 DISCUSSION

Bone is an active tissue, undergoing continuous remodelling by orchestrated processes of osteoclastic bone resorption and osteoblastic bone formation.^33^ Various conditions, such as osteoporosis, fracture and rheumatoid arthritis, occur because of loss of normal bone structure, function and homoeostasis.^1^ BMPs have widely recognized roles in bone formation during mammalian development, rendering them promising candidates for therapeutic use.^34^ However, side effects such as immunogenic response and increased cancer risk associated with higher BMP doses, as well as pro‐adipogenic effects,^35^ hinder its clinical application in treating bone disorders. Therefore, titrating the BMP dose and blocking its pro‐adipogenic effects could advance BMP‐based therapy for bone diseases. This study demonstrates that Lox inhibition greatly enhances osteogenesis at lower doses of BMP4, and suppresses pro‐adipogenic effects of BMP4 synchronously. Lox inhibition greatly up‐regulates BMP4‐induced expression of CHOP‐10, which favours the basal and BMP4‐induced osteogenesis while disrupting adipogenesis. These effects are mediated through inactivation of p38 MAPK and Smad signalling, and activation of Wnt/β‐catenin signalling. We further demonstrate the reciprocal regulation between Lox and CHOP‐10. Collectively, we identify a novel therapeutic targeting Lox in obesity and bone disorders.

A complicated and controversial relationship exists between obesity and bone health. BMP4 function in bone is complex.^36^ Recently, it has been demonstrated that increased circulating levels of BMP4 in obese human subjects and diet‐induced obesity (DIO) mice,^37^, ^38^ which is often accompanied with osteoporosis, suggesting a osteoporosis promotion role of BMP4. Furthermore, disruption of signalling through BMPR1A in adult osteoblasts or osteoclasts^39^, ^40^, ^41^ increases bone mass provides evidence that alteration of the physiologic levels of BMPs and/or altering BMPR1A may have therapeutic effects on bone loss in vivo. Our findings reveal that Lox inhibition promotes BMP4‐induced osteogenesis, which contradicts previous reports about the functions of Lox in bone development.^14^, ^42^, ^43^, ^44^ Specifically, primary calvarial osteoblasts from Lox^−/−^ mice exhibit decreased osteoblastic differentiation,^14^ and BAPN inhibits osteoblastic differentiation of MC3T3‐E1 preosteoblasts.^44^, ^45^ This discrepancy may be owing to the presence of BMP4, a molecule with both pro‐adipogenic and pro‐osteogenic effect, in that BAPN alone inhibited osteogenesis both in vitro (Figure S1) and in vivo (Figure S5), which was consistent with the previous study that Lox inhibit osteoblast differentiation.

We have previously reported that BMP4 promotes adipocyte lineage commitment at a concentration that can also promote the osteogenic lineage commitment found in this study. Here, we found that Lox inhibition greatly enhanced BMP4‐induced expression of CHOP‐10 (Figures 2A‐C and S2), which jeopardizes the pro‐adipogenic effects of BMP4 (Figure S3C,D) and further advances its pro‐osteogenic effects (Figures 2D‐F and S3A). Moreover, overexpression of Lox in C3H10T1/2 stem cells depresses both BMP4 and CHOP‐10 induced osteogenesis (Figure 2G‐I), while accelerating adipogenesis (Figure S3E,F). Of note, BMP4 was significantly increased in bone of FABP‐4 driven BMP4‐Tg mice, the Lox inhibitor BAPN also enhanced bone formation in BMP4‐Tg mice in vivo (Figure 4). Therefore, BAPN in conjunction with BMP4 prevents adipogenesis while accelerating BMP4‐induced osteogenesis, presenting a potential avenue to ameliorate the detrimental effects of BMP4 while preserving its beneficial actions. In general, we found that Lox inhibition facilitates BMP4 and CHOP‐10 induced osteoblast lineage commitment by impeding adipocytic lineage commitment of MSCs.

It has been reported that *Chop* null mice exhibit decreased bone formation, indicating that CHOP‐10 is crucial for osteoblastic function in vivo.^46^ Here, we have demonstrated that CHOP‐10 stimulates both basal and BMP4‐induced osteogenesis (Figure 2D‐F and S3A), in agreement with previous reports using ST‐2 stromal cells.^24^ However, a previous study showed impaired osteoblastic function and osteopenia in transgenic mice overexpressing CHOP‐10 because of increased osteoblast apoptosis.^47^ We also found that CHOP‐10 promotes BMP4‐induced osteogenesis by hampering commitment to the adipocyte lineage of MSCs (Figure S3B,C). Additionally, we identified a reciprocal regulation between Lox and CHOP‐10 (Figure 2G‐I), providing possibilities for precisely regulating CHOP‐10 so as to balance adipogenic and osteogenic differentiation. Generally, CHOP‐10 is necessary for normal osteoblast differentiation, but when in excess and under specific conditions in vivo, it could be detrimental to bone homoeostasis.

The effects of Wnt/β‐catenin signalling on bone mass have been well‐established in both mouse models and human patients,^48^ showing both pro‐osteogenic and anti‐adipogenic effects.^6^ In addition, there is an intricate crosstalk between Wnt/β‐catenin and BMPs, both functioning as master regulators of osteogenesis. BMPs, in combination with Wnt, induce MSCs to commit to osteoblastic lineage and enhance the pool and function of mature osteoblasts. Accordingly, BMP antagonists like Gremlin bind and suppress BMP signalling and activity in osteoblastic lineage cells, which tempers Wnt signalling,^49^ whereas deletion or down‐regulation of Gremlin sensitizes osteoblastic cells to the actions of BMP and Wnt.^50^ Our data show that Wnt/β‐catenin signalling is activated to stimulate BMP4‐induced osteogenesis by Lox inhibition and CHOP‐10 overexpression (Figures 3 and S4). However, CHOP‐10 has also been reported to inhibit Wnt/TCF signals in response to Wnt‐8 in human embryonic and colon cancer cell lines.^51^ This contradiction may be because of how the function and regulation of CHOP‐10 varies among different cell lines and models studied.

The impact of obesity on bone health has been controversial for a long time. Obesity was traditionally viewed to be beneficial to bone health.^52^ However, increasing evidence has shown that obesity could be a risk factor for osteoporosis.^52^, ^53^ Here, we showed a significant bone loss in HFD‐induced obesity mice (Figure 5), in line with reports that obesity is associated with bone loss.^52^ We also discovered an inverse correlation between Lox and bone markers in both NCD and HFD mice (Figure 5). We detected higher expression of Lox, lower expression of CHOP‐10 and Ocn and decreased bone formation in HFD mice femurs (Figure 5D), in line with reports that obesity is associated with decreased bone mass.^52^ However, the mechanism underlying how Lox up‐regulates CHOP‐10 expression is yet to be uncovered.

In general, our study uncovered a novel Lox/CHOP‐10 crosstalk governing the osteogenic and adipogenic cell decisions of MSCs. We demonstrated that Lox inhibition greatly enhances BMP4‐induced osteogenesis, while suppressing the pro‐adipogenic effect of BMP4 (Figure 5E). This could advance the clinical use of BMPs in bone diseases. Our study also provides a new path towards advancing the fundamental understanding of reciprocal connections between fat and bone tissue, presenting Lox as a promising target in prevention of bone and fat diseases.

## CONFLICT OF INTEREST

The authors confirm that there are no conflicts of interest.

## AUTHOR CONTRIBUTIONS

Wenyan Jiang performed conception and design, collection and assembly of data, data analysis and interpretation, manuscript writing; Chun Xing performed animal studies; Hong‐wei Wang, Wei Wang contributed to provision of study material; Su‐zhen Chen, Liu‐fang Ning, Xu Xu, Qi‐qun Tang performed data analysis and interpretation; Hai‐yan Huang performed conception and design, data analysis and interpretation, financial support, manuscript writing, final approval of manuscript.
