# Supplementary material for: A Lox/CHOP‐10 crosstalk governs osteogenic and adipogenic cell fate by MSCs
Source: J Cell Mol Med. 2018 Jul 25;22(10):5097–108. doi: 10.1111/jcmm.13798 (PMC6156357; doi:10.1111/jcmm.13798)
Supplement: Supplementary file 1 [file JCMM-22-5097-s001.doc]

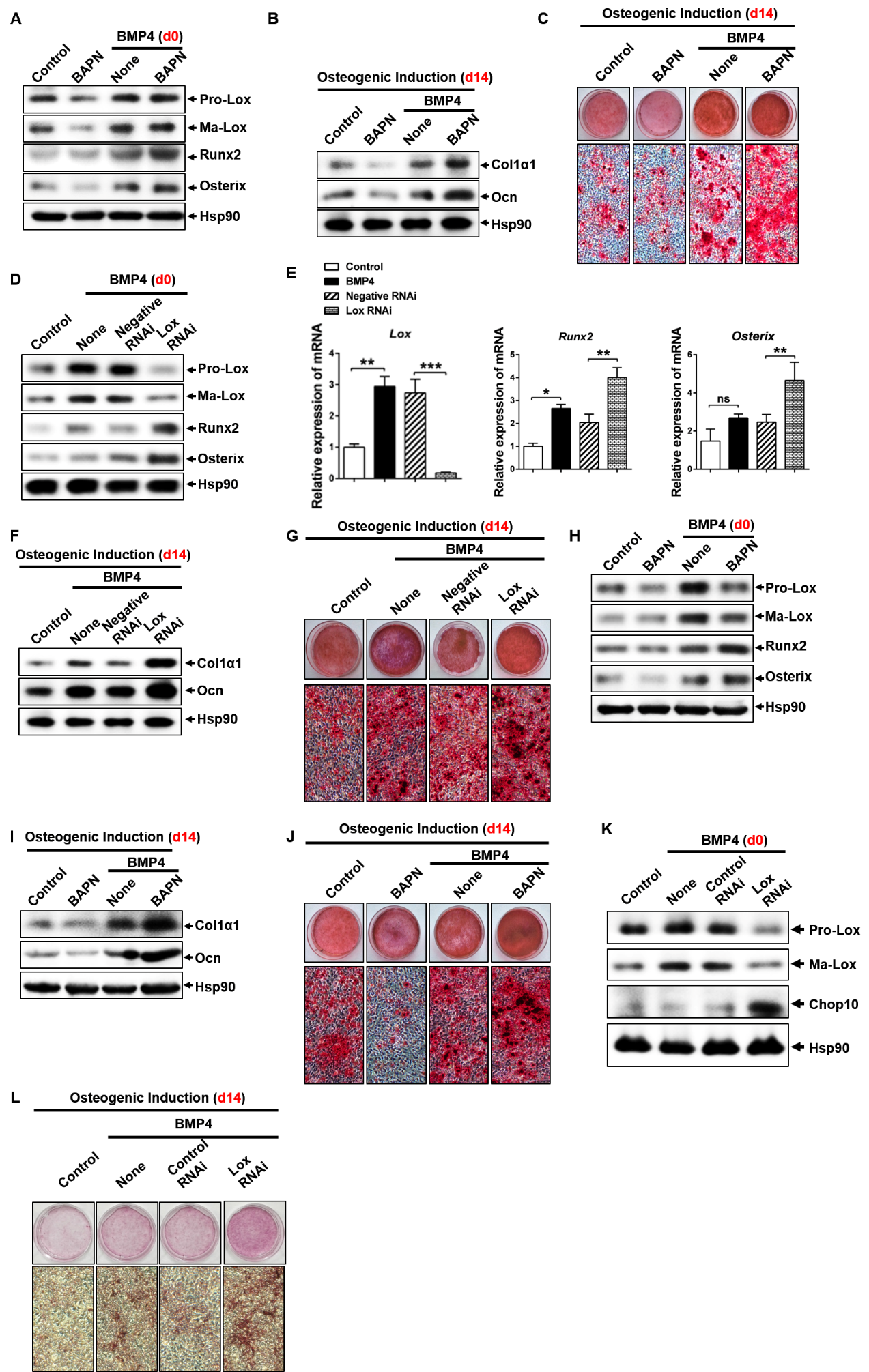
**Supplemental data**

**Figure S1. Lox inhibition promotes BMP4-induced osteogenesis.**

(A-C) The effect of BAPN (200 µM) on BMP4-induced osteoblast lineage commitment of C3H10T1/2 cells and subsequential differentiation was identified by Runx2 and Osterix expression on day 0 (A) Col1α1 and Ocn expression (B) and Alizarin Red S staining (C) on day14.

(D and E) The effects of Lox knockdown on BMP4-induced osteoblast lineage commitment of SVFs cells on day 0 were assessed by western blotting (D) and Q-PCR (E).

(F and G) The effects of Lox knockdown on BMP4-induced osteogenic differentiation of SVFs cells on day 14 was evaluated by western blotting (F) Alizarin Red S staining (G).

(H-J) The effect of BAPN (200 µM) on BMP4-induced osteoblast lineage commitment of SVFs and subsequential differentiation indicated by Runx2 and Osterix expression on day 0 (H) and Col1α1 and Ocn expression (I) and Alizarin Red S staining (J)on day14.

(K) The effects of Lox knockdown on CHOP-10 expression during BMP4-induced osteoblast lineage commitment of BMSCs were assessed by western blotting.

(L) The effects of Lox knockdown on BMP4-induced osteogenic differentiation of BMSCs on day 14 was evaluated by Alizarin Red S staining.

*P < 0.05, **P < 0.01, ***P < 0.001, ****P < 0.0001.


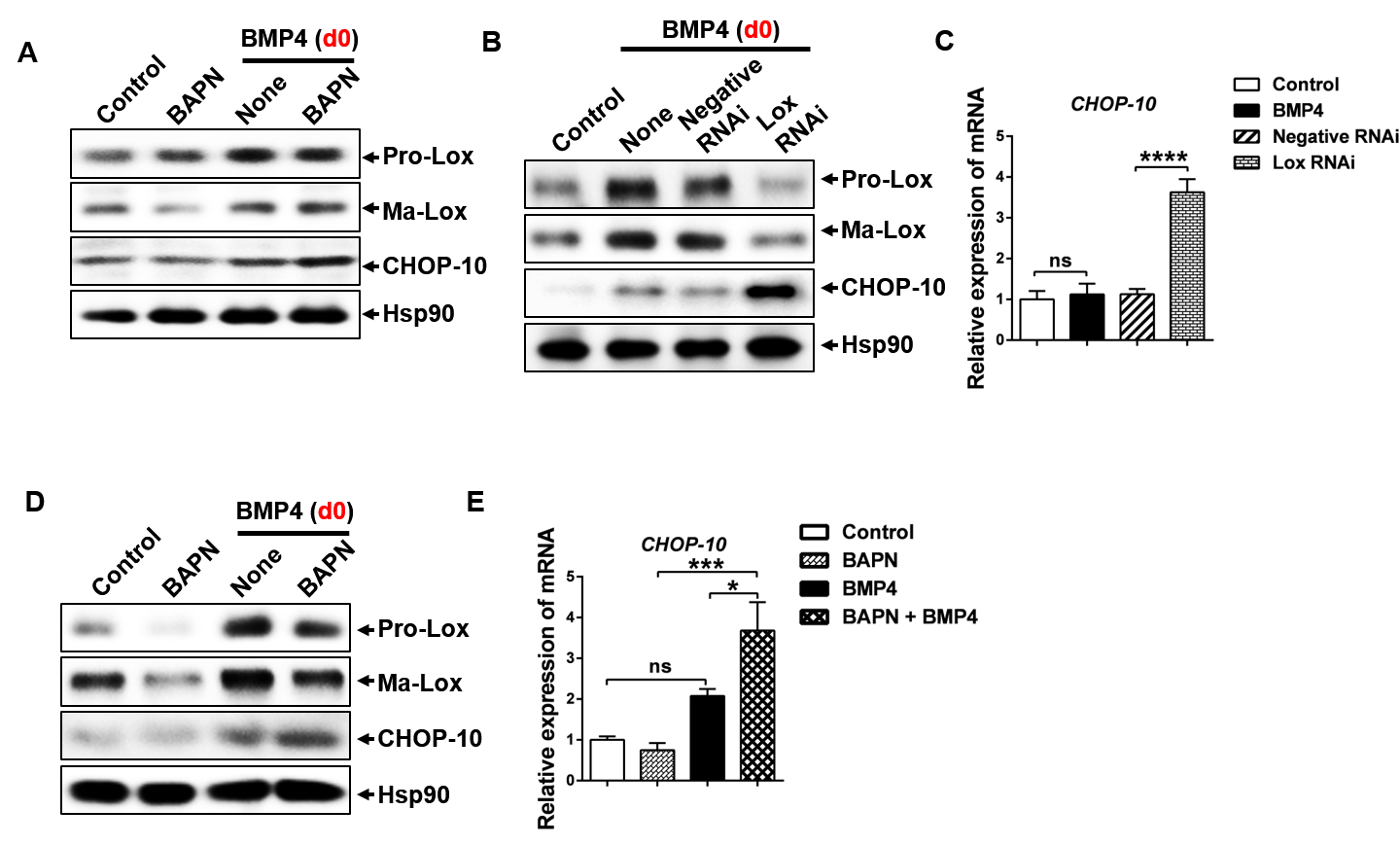


**Figure S2. Lox inhibition up-regulates BMP4-induced expression of CHOP-10.**

(A)The effect of the Lox inhibitor BAPN on the expression of CHOP-10 in C3H10T1/2 at the committed stage was confirmed by western blotting.

(B and C) Lox knockdown and its effect on CHOP-10 expression at the committed stage (day 0) of SVFs from inguinal adipose tissue was assessed by western blotting (B) and Q-PCR (C).

(D and E) The effect of the Lox inhibitor BAPN on the expression of CHOP-10 at the committed stage (day 0) of SVFs from inguinal adipose tissue was confirmed by western blotting (D) and Q-PCR (E) at the committed stage (day 0).


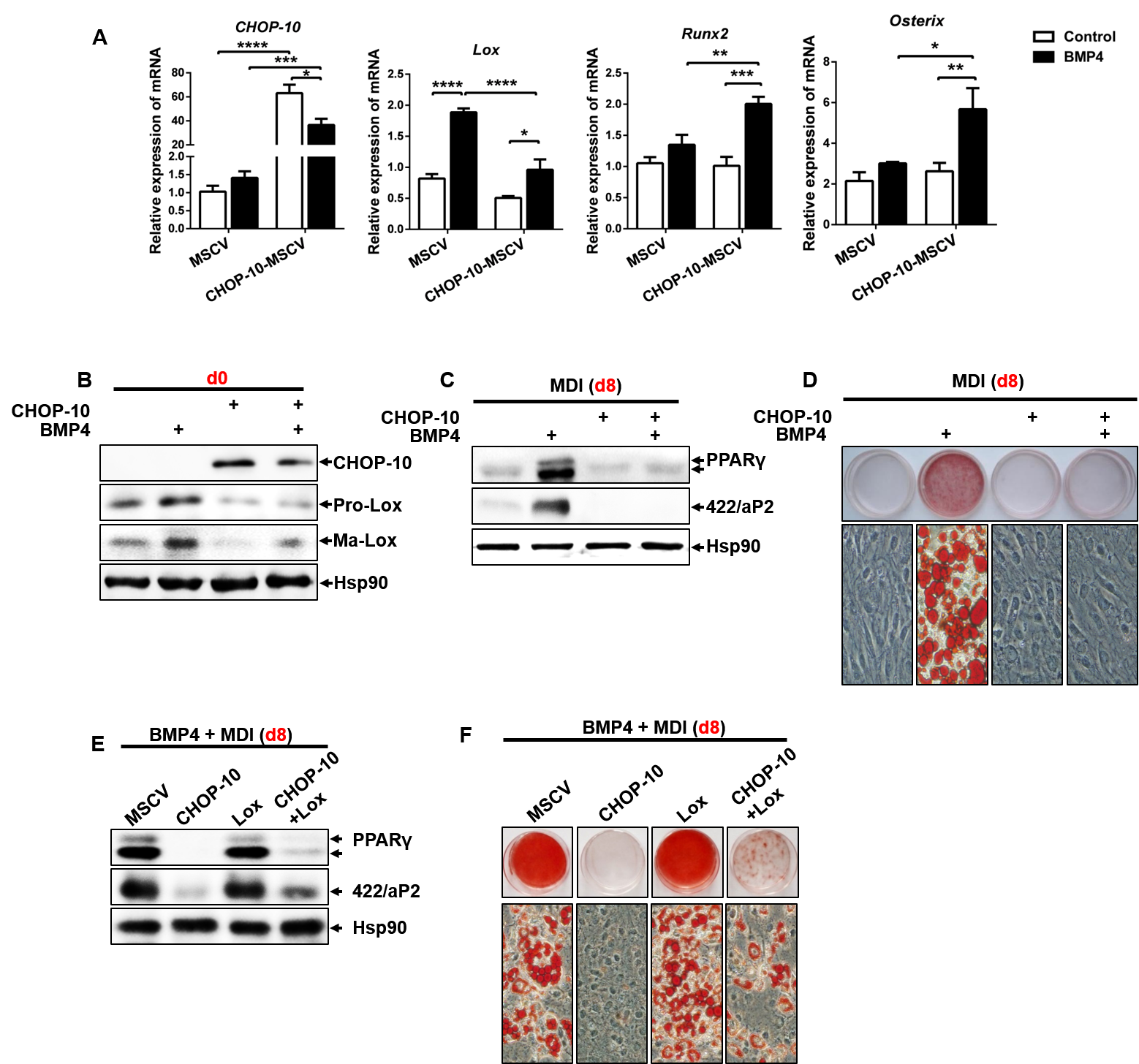


**Figure S3. Lox rescues adipogenic differentiation inhibited by CHOP-10**

C3H10T1/2 stem cells transfected with indicated retrovirus were treated with or without BMP4 until post-confluence (Day 0). Then, the 2-day post-confluent cells were induced to adipocyte differentiation using the standard MDI protocol as described in Materials and Methods.

1. Osteoblast-specific markers at the committed stage were detected by Q-PCR.

(B-D) Expression of CHOP-10 and Lox was confirmed by western blotting (B) at the committed stage (day 0). The effect of CHOP-10 overexpression on adipogenesis was assessed by expression of PPARγ, 422/aP2 (C), and Oil Red O staining (D) on day 8. CHOP-10: CCAAT/enhancer binding protein (C/EBP) homologous protein 10.

(E) Expression of PPARγ and 422/aP2 on day 8 after adipogenic induction.

(F) Lipid droplets stained by Oil Red O on day 8 after adipogenic induction.


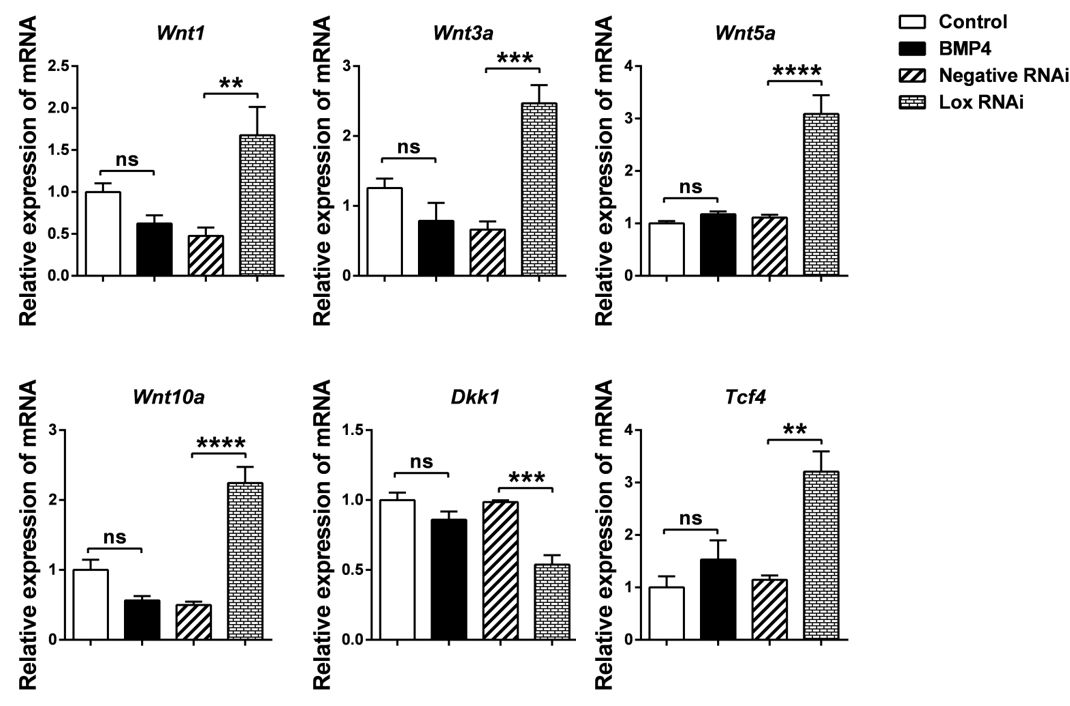


**Figure S4. The effect of Lox knockdown on the expression of Wnt signaling molecules.**

SVF cells from inguinal adipose tissue were transfected with Lox RNAi or negative RNAi in the presence of BMP4. Knockdown efficiency and its effect on the expression of Wnt signaling molecules were detected by Q-PCR.


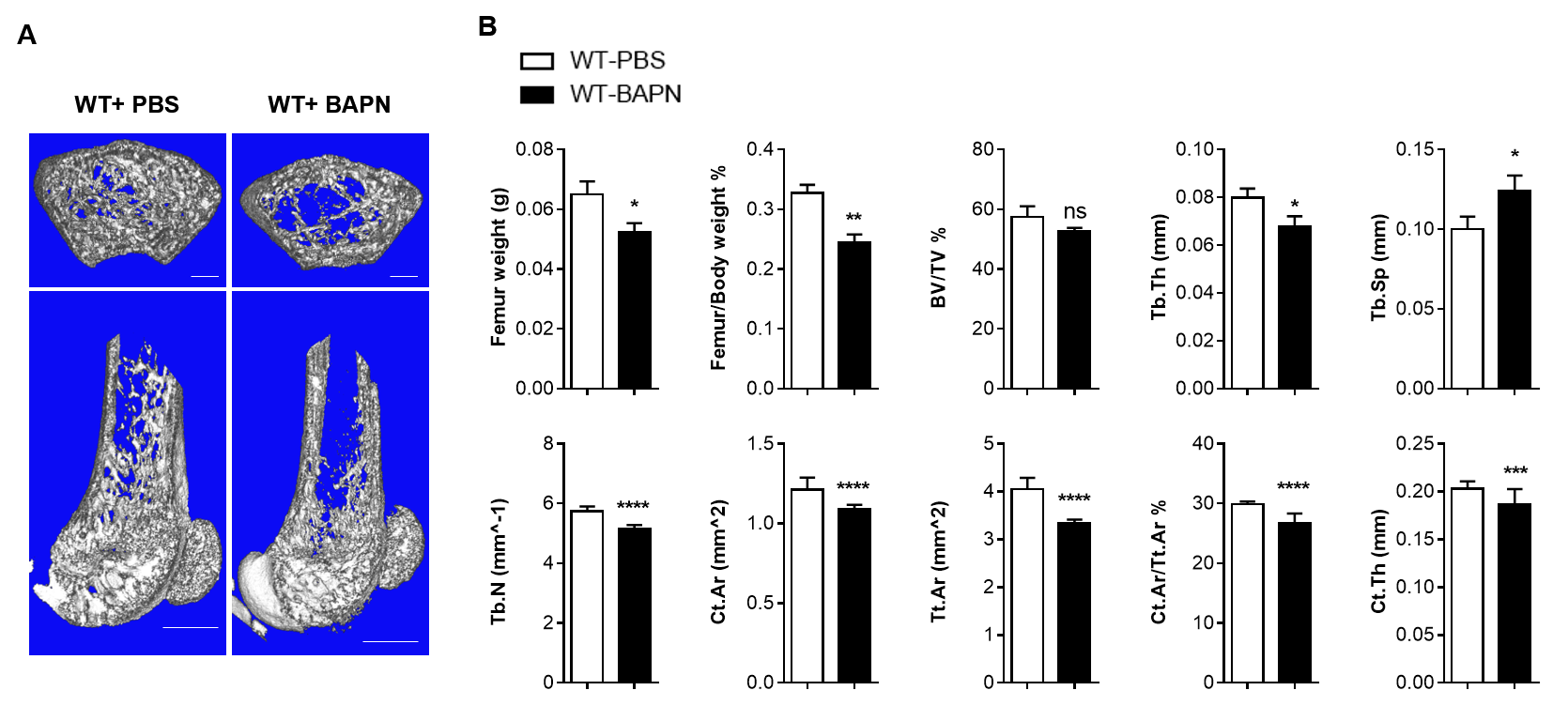


**Figure S5. Injection of BAPN alone inhibits bone formation *in vivo*.**

(A) 3D micro-CT images of cross sections (upper panel) and vertical sections (lower panel) of right femur extracted from WT mice with PBS or BAPN treatment. Bars: upper panel, 200 µm; lower panel, 1 mm.

(B) Quantification of trabecular and cortical bone for the same femur as A.
